# Molecular Analysis of Microbial Communities in Endotracheal Tube Biofilms

**DOI:** 10.1371/journal.pone.0014759

**Published:** 2011-03-14

**Authors:** Scott Cairns, John Gilbert Thomas, Samuel James Hooper, Matthew Peter Wise, Paul John Frost, Melanie Julia Wilson, Michael Alexander Oxenham Lewis, David Wynne Williams

**Affiliations:** 1 University Hospital of Wales, Cardiff, United Kingdom; 2 West Virginia University, Morgantown, West Virginia, United States of America; 3 School of Dentistry, Cardiff University, Cardiff, United Kingdom; University of Hyderabad, India

## Abstract

**Background:**

Ventilator-associated pneumonia is the most prevalent acquired infection of patients on intensive care units and is associated with considerable morbidity and mortality. Evidence suggests that an improved understanding of the composition of the biofilm communities that form on endotracheal tubes may result in the development of improved preventative strategies for ventilator-associated pneumonia.

**Methodology/Principal Findings:**

The aim of this study was to characterise microbial biofilms on the inner luminal surface of extubated endotracheal tubes from ICU patients using PCR and molecular profiling. Twenty-four endotracheal tubes were obtained from twenty mechanically ventilated patients. Denaturing gradient gel electrophoresis (DGGE) profiling of 16S rRNA gene amplicons was used to assess the diversity of the bacterial population, together with species specific PCR of key marker oral microorganisms and a quantitative assessment of culturable aerobic bacteria. Analysis of culturable aerobic bacteria revealed a range of colonisation from no growth to 2.1×10^8^ colony forming units (cfu)/cm^2^ of endotracheal tube (mean 1.4×10^7^ cfu/cm^2^). PCR targeting of specific bacterial species detected the oral bacteria *Streptococcus mutans* (n = 5) and *Porphyromonas gingivalis* (n = 5). DGGE profiling of the endotracheal biofilms revealed complex banding patterns containing between 3 and 22 (mean 6) bands per tube, thus demonstrating the marked complexity of the constituent biofilms. Significant inter-patient diversity was evident. The number of DGGE bands detected was not related to total viable microbial counts or the duration of intubation.

**Conclusions/Significance:**

Molecular profiling using DGGE demonstrated considerable biofilm compositional complexity and inter-patient diversity and provides a rapid method for the further study of biofilm composition in longitudinal and interventional studies. The presence of oral microorganisms in endotracheal tube biofilms suggests that these may be important in biofilm development and may provide a therapeutic target for the prevention of ventilator-associated pneumonia.

## Introduction

Ventilator-associated pneumonia (VAP) is the most frequent nosocomial infection in the intensive care unit (ICU) occurring in 8–28% of mechanically ventilated patients [1], [2]. Mortality rates are high (15–70%) and length of stay is increased, adding a cost of approximately $40,000 per patient [3], [4]. The presence of an endotracheal tube is an independent risk factor for developing VAP and whilst tracheal intubation is necessary to facilitate mechanical ventilation, it also circumvents elements of patients' innate immunity. The endotracheal tube disrupts the cough reflex, promotes accumulation of tracheobronchial secretions and mucus, and provides a direct conduit for pathogenic microorganisms to reach the lower respiratory tract, increasing the risk of infection [5]. Significantly, the endotracheal tube may also act as a reservoir for pathogens by providing a surface to which they can adhere and form biofilms [5], [6], [7]. Polymicrobial biofilms develop rapidly following intubation along the inner lumen of the endotracheal tube, with well-organised antibiotic-resistant structures detectable within twenty-four hours [6], [7]. Furthermore, 70% of patients with VAP have been reported as having identical pathogens present within the endotracheal tube biofilm as encountered in the lung [8], suggesting that the biofilm represents a significant and persistent source of pathogenic bacteria.

There is mounting evidence to support the concept that poor oral health is related to the aetiology of bacterial VAP and a number of studies have suggested improved oral hygiene may be effective at reducing its incidence [9]-[12]. Moreover, potential respiratory pathogens such as *Pseudomonas aeruginosa* and *Staphylococcus aureus*, that are not generally regarded as normal inhabitants of the oral microflora, are isolated more frequently in the dental plaque of ICU patients than that of the general population [13], [14]. Given that these potentially pathogenic bacteria are capable of adhering to and forming biofilms with the microflora of the oral cavity (*i.e.* dental plaque), it seems plausible that oral plaque bacteria may also be present in biofilms of endotracheal tubes. The presence of oral microorganisms in a biofilm within endotracheal tubes may have significance beyond facilitating the adherence of potential respiratory pathogens. Co-aggregation of different microbial species can enhance the virulence characteristics of certain bacteria, as well increasing their tolerance to antimicrobials [15].

The composition of biofilms in general [16], and those originating in the oral cavity, in particular, is often misrepresented by traditional cultural analysis [17]. This distortion is apparent even for microorganisms amenable to culture, since recovery from a biofilm environment is subject to many biases including the ability of species to ‘out-compete’ neighbouring species in a given environment and variable replication times. The oral microflora is the most diverse bacterial community in the human body with over 700 species present, and cultural analysis is complex. In addition, given that over 50% of the oral microflora is considered to be unculturable or, more correctly, ‘not-yet-cultured’ the community composition is likely to be considerably distorted by cultural analysis [17].

Numerous culture independent approaches are available for the analysis of mixed microbial communities. One of the most useful has proven to be the community profiling technique known as denaturing gradient gel electrophoresis (DGGE), a method used extensively by microbial ecologists for the study of environmental communities [18], [19]. This approach has been successfully applied to the analysis of wounds, corneal ulcers, gastrointestinal tract and oral cavity [20]-[24]. DGGE is based on the PCR amplification of phylogenetically useful molecules (typically 16S ribosomal DNA) from mixed bacterial communities and the subsequent separation of the amplicons on a denaturing electrophoretic gel. The conditions allow the separation of bands of the same length that have different nucleotide composition. The resulting banding profiles can be analysed to reveal differences in the predominant bacterial composition of samples in cross-sectional and longitudinal studies.

The aim of this study was to characterise microbial biofilms on the inner luminal surface of extubated endotracheal tubes from ICU patients. More specifically the presence of oral marker bacteria and VAP associated pathogens was determined by PCR and the usefulness of DGGE community profiling for the analysis of the endotracheal tube biofilm was assessed.

## Materials and Methods

### Collection and processing of endotracheal tubes

A total of 24 extubated endotracheal tubes were obtained from 20 patients (12 male and 8 female; age range 20–79 years; mean 61 years) who were intubated and mechanically ventilated on the ICU of a university teaching hospital. Two endotracheal tubes were obtained from each of two of these patients and one patient provided three endotracheal tubes. The duration of intubation prior to endotracheal tube collection was 12–289 h (mean 92 h). Prior to the study the South East Wales IRB confirmed that the project raised no ethical issues and therefore informed consent was not required.

Collected endotracheal tubes were placed in a sealed sterile bag and transferred immediately to the microbiology laboratory for processing (undertaken within 1 h of endotracheal tube collection). From the central region of each endotracheal tube two 1 cm sections were cut and processed. One was used for quantitative microbial culture and the other for molecular analysis. The biofilm was aseptically removed from the endotracheal tube lumen using a sterile scalpel and this was resuspended in 2 ml of phosphate buffered saline (PBS). The preparation was vortex mixed for 30 s with sterilised glass beads to disrupt any biofilm aggregates.

### Quantitation of microbial colonisation of the endotracheal tube

The PBS solution containing recovered biofilm was serial decimally diluted 10^6^-fold. Using a spiral-plater system (Don Whitley Scientific, Shipley, UK), 50 µl volumes of each dilution was deposited on to agar media. Quantitation of aerobic microorganisms was achieved by culture on blood agar (BA; LabM, Bury, UK) whilst CHROMagar® *Candida* (CHROMagar, Paris, France) was used to detect *Candida*. Inoculated agars were incubated for 37 °C for 48 h. A total aerobic microbial count from the sections was obtained and expressed as total colony forming units (cfu) per cm^2^ of endotracheal tube section. In addition, *Candida* species were identified based on colony colour and appearance on CHROMagar® *Candida* [25] and by biochemical profiling using the Auxacolor 2 system (Bio-Rad, Hemel Hempstead, UK) [26].

### 16S ribosomal RNA gene-defined bacterial colonisation of endotracheal tubes

Sections from each region of the endotracheal tubes were analysed using molecular procedures. The method involved the extraction of total DNA from 500 µl of the PBS specimens using a Puregene® bacterial DNA isolation kit (Qiagen). A universal bacterial primer pair, 341F and 534R [27] was then used to amplify the 16S rRNA gene targets within the extracted samples using standard PCR and reaction conditions. Negative controls included a reagent control (sterile water served as PCR template) and a sample preparation control (sterile water used in place of the original sample and exposed to the entire extraction protocol). Positive controls of template DNA from three known species *Porphyromonas gingivalis* NCTC 11834^T^, *Staphylococcus aureus* NCTC 6571 and *Streptococcus mutans* NCTC 20523^T^) were also included. PCR products were then resolved by denaturing gradient gel electrophoresis (DGGE) as outlined below with the products from the control microorganisms serving as markers to facilitate gel normalisation.

### Denaturing Gradient Gel Electrophoresis

Polyacrylamide gels were cast using a model 385 gradient former (Bio-Rad). All gels comprised of 1×Tris-acetate-EDTA (TAE) buffer with 10% (w/v) acrylamide, 0.1% (v/v) TEMED, and 0.1% (w/v) ammonium persulphate (all materials from Sigma, Poole, UK). Gels also contained a parallel 30–60% gradient of denaturants (where 100% denaturant concentration was equal to 7 M urea and 40% [v/v] deionised formamide; Sigma).

Parallel DGGE was performed using the Bio-Rad D-Code system. Products from endotracheal tube extract PCRs were run alongside a marker comprising of an equal mixture of PCR products from the three known bacterial stains. DGGE was run in 1×TAE buffer at 56 °C and 160 V/cm^2^ for 30 min, followed by 40 V/cm^2^ for 16 h.

DGGE gels were stained with SYBR® Green I nucleic acid gel stain (Sigma) at approximately 4 °C for 30 min. Banding patterns were visualised under UV light using a GelDoc system (Bio-Rad). Gels were then aligned and standardised using the reference markers and GelCompar II software (Applied Maths, Sint-Martens-Latem, Belgium). The presence of individual bands in each profile was assessed using the same software, set to a 1% tolerance of band matching. Using Dice's coefficient and the UPGMA clustering method, a dendrogram of genetic similarity between samples was calculated.

### Detection of bacteria by species-specific PCR

Species-specific detection of the oral marker organisms *S. mutans* and *P. gingivalis*, together with the accepted VAP causing organisms *P. aeruginosa* and *S. aureus* was also undertaken according to previously published protocols [28]-[31].

## Results

### Culture of microorganisms from biofilms recovered from endotracheal tubes

The results of microbial culture are summarised in Table 1. In the case of five endotracheal tube samples, no microbial growth was observed. The viable bacterial aerobic counts ranged between 2×10^4^ and 2.1×10^8^ cfu/cm^2^ of endotracheal tube section. In 5 endotracheal tubes no viable aerobic bacterial growth was detected, although in one of these tubes *Candida* was cultured. There was no apparent relationship between the determined bacterial load on the sample and the duration of intubation. In six endotracheal tubes from five patients, *C. albicans* was isolated following subculture on CHROMagar®*Candida* and biochemical identification.

**Table 1:** Band numbers obtained by DGGE analysis, PCR and culture results for selected organisms.

			DGGE	PCR detection				Culture	
Patient number	Endotracheal tube	Hours intubated	Number of bands	*S. mutans*	*P. gingivalis*	*P. aeruginosa*	*S. aureus*	*C. albicans*	Total aerobic bacterial count cfu/cm^2^ endotracheal tube
1	1	33	5						2.3×10^7^
2	2	108	16	+				+	6.3×10^6^
2	7	94	4						2.8×10^7^
3	3	60	17					+	1.8×10^6^
4	4	95	14			+			6.1×10^6^
5	5	43	22					+	2.0×10^4^
6	10	41	9		+		+		0
7	8	289	8						3.0×10^7^
8	9	173	15	+		+	+	+	4.0×10^4^
9	11	198	10				+		2.1×10^8^
10	12	56	6		+				2.0×10^6^
11	13	137	5						7.7×10^6^
12	14	12	3						4.0×10^4^
12	15	20	5					+	0
12	16	96	6	+				+	1.6×10^5^
13	17	224	4						0
14	18	49	5		+		+		0
15	19	29	3						4.0×10^4^
16	20	67	7	+			+		1.3×10^7^
17	21	20	5			+			0
17	24	143	5			+			7.7×10^6^
18	22	76	4		+		+		1.4×10^7^
19	23	32	9	+	+				1.8×10^5^
20	25	125	3						8.2×10^6^

### DGGE estimation of species richness

**Figure 1:**
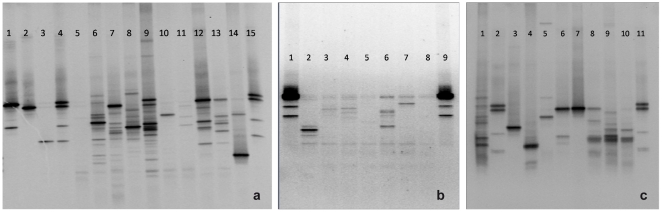
DGGE profiles of PCR amplified 16S rRNA from marker bacteria and endotracheal tube (ETT) biofilm samples. **Figure 1a:** 1, *P. gingivalis*; 2, *S. aureus*; 3, *S. mutans*; 4, mixed marker of *P. gingivalis*, *S. aureus* and *S. mutans*; 5, ETT1; 6, ETT2; 7, ETT3; 8, ETT4; 9, ETT5; 10, ETT7; 11, ETT8; 12, ETT9; 13, ETT10; 14, ETT11; 15, mixed marker of *P. gingivalis*, *S. aureus* and *S. mutans*. **Figure 1b:** 1, mixed marker of *P. gingivalis*, *S. aureus* and *S. mutans*; 2, ETT12; 3, ETT14; 4, ETT15; 5, ETT19; 6 ETT21; 7, ETT25; 8, ETT26; 9, mixed marker of *P. gingivalis*, *S. aureus* and *S. mutans*. **Figure 1c:** 1, Plaque sample control; 2, *mixed marker of P. gingivalis, S. aureus and S. mutans*; 3, ETT13; 4, ETT16;5, ETT17; 6, ETT18; 7, ETT20; 8, ETT22; 9, ETT23; 10, ETT24; 11, mixed marker of *P. gingivalis, S. aureus and S. mutans*.

**Figure 2:**
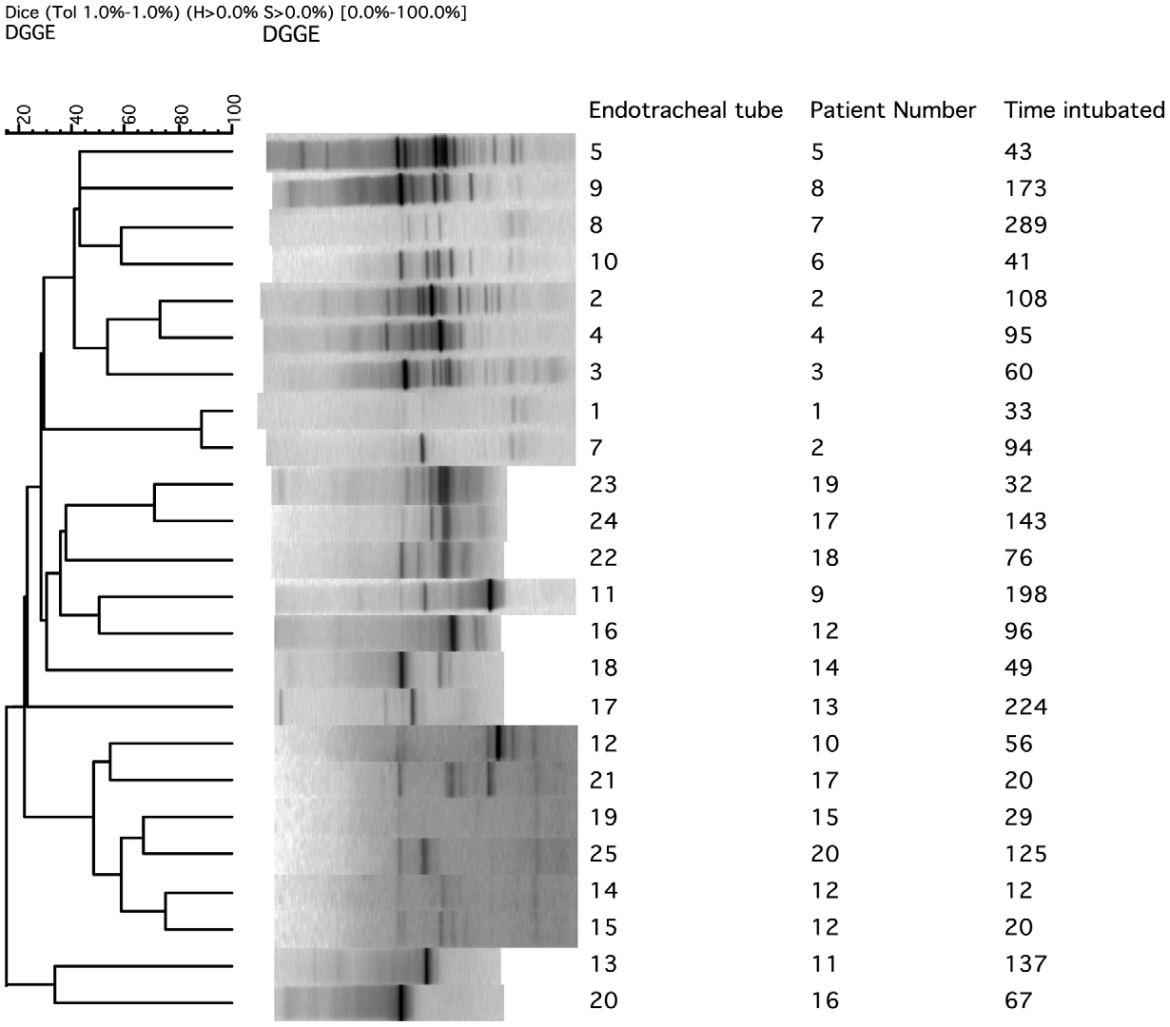
Cluster analysis with Ward's algorithm based on the Dice coefficient demonstrating the diversity in DGGE profiles generated from endotracheal tube (ETT) biofilms.

The number of bands obtained by DGGE analysis is shown in Table 1 and typical profiles are presented in Figure 1. An average of 6 bands (range 3 to 22) per endotracheal tube sample was detected and the similarity in banding profiles ranged between 20 and 90% (Fig. 2). There was no apparent relationship between the duration of intubation and the number of DGGE bands detected, nor was there a high level of similarity for the DGGE profiles obtained for multiple endotracheal tubes from the same patients (*i.e.* patient 2, tubes 2 and 7; patient 17, tubes 21 and 24). However, similarity at 75% was evident for the profiles of two endotracheal tubes (14 and 15) which were obtained from patient 12 (Fig. 2). DGGE was shown to be reproducible with consistent profiles being generated upon both repeat and duplicate testing. The community profiles obtained demonstrated considerable inter-patient and intra-patient diversity.

### Detection of marker bacteria by species specific PCR

**Figure 3:**
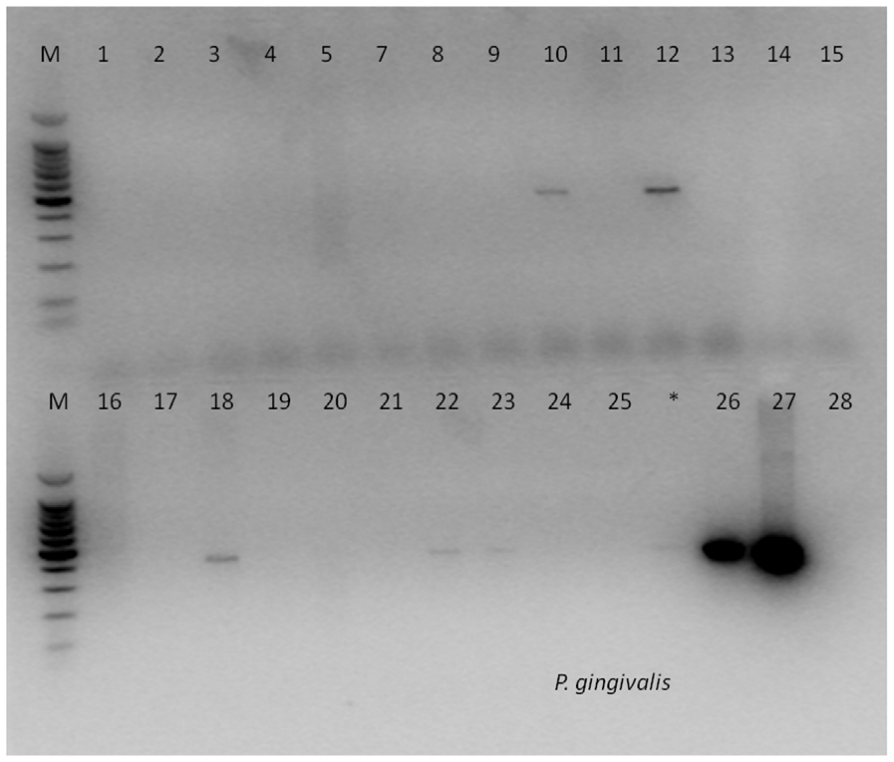
Result of the *P. gingivalis*-specific PCR using template DNA obtained from the biofilms recovered from endotracheal tubes 1 – 25. Lane 26 contains the result from a dental plaque sample known to contain *P. gingivalis*, used here as a positive control. Lane 27 is a positive control using template DNA from *P. gingivalis* NCTC 11834^T^. Lane 28 is the template-free negative control.

At least one of the oral marker bacterial species (*S. mutans* or *P. gingivalis*) was detected by PCR for 9 different patients. Of these 9 patients, PCR was positive only for *S. mutans* in 4 patients, as was the case for *P. gingivalis*, the endotracheal tube from one patient (Patient 19, endotracheal tube 23) was positive for both oral bacterial species (Table 1). Figure 3 shows a typical result of the *P. gingivalis* specific PCR using template DNA obtained from the biofilms recovered from the endotracheal tubes. The detection of the VAP associated species *P. aeruginosa* and *S. aureus* occurred in 4 and 6 patients, respectively. The patient distribution of these microorganisms is presented in Table 1.

## Discussion

Sottile and colleagues first suggested a link between the endotracheal biofilm and pulmonary infection [32], and this was supported by identical pathogenic bacteria being present in the lung of patients with VAP and the endotracheal biofilm [8]. This concept has been substantiated by a number of experimental and clinical studies, which have used antimicrobial (silver coated) endotracheal tubes to inhibit biofilm formation [33]-[36], reduce colonisation of the airway [33]-[36] and decrease the incidence of VAP [37]. The reduction in the number and delay of onset of VAP in a large clinical trial is encouraging, however, bacteria can develop silver resistance [38] and other strategies, which target biofilm formation, may prove to be valuable. In order to prevent endotracheal tube biofilm formation, knowledge of the source of the microorganisms involved is essential.

Improved oral hygiene has proved to be an effective strategy for reducing VAP [9]-[12] and since potential respiratory pathogens are isolated from dental plaque (a biofilm) of mechanically ventilated patients [13], [14], we hypothesised that microbes which constitute the normal oral flora might be present in the endotracheal biofilm. In an *in vitro* Ventilator-Endotracheal-Lung model incorporation of representative species of the oral microflora prior to the addition of pathogens traditionally associated with respiratory infection, augments the subsequent biofilm (JG Thomas, unpublished data), raising the possibility that normal oral microflora may represent pioneering colonising species and promote subsequent endotracheal tube biofilm development. The inner surface of the endotracheal tube provides a unique environment of high shear forces, bi-directional flow, and a restricted immunological environment, which favours a sessile rather than planktonic bacterial life form and thus biofilm formation. The endotracheal biofilm is a complex three-dimensional structure with cyclical bacterial communities many of which are difficult to isolate by conventional planktonic culture techniques [39]. Bacterial species regarded as appropriate markers of the oral microflora (*P. gingivalis* and *S. mutans*) were detected in 9 of the 20 patients using species specific PCR. *Streptococcus mutans* was selected as a target species due to its recognised ability to promote biofilm formation, whilst *P. gingivalis* was included as a representative species of strictly anaerobic bacteria.

Interestingly, *Candida albicans*, which is also a common coloniser of the oral cavity, was detected by culture in the endotracheal tubes from 5 patients. This yeast produces filamentous growth forms, which could readily enhance formation and structural stability of the biofilm [40]. Interactions between individual elements of the oral microflora and their capacity to form biofilms are complex. *Streptococcus mutans* enables *C. albicans* to bind to synthetic surfaces, coaggregation being dependent on the production of water insoluble glucans and direct cell-cell contact [41], [42], [43]. *Candida albicans* also demonstrates synergistic and antagonistic interactions with *S. aureus* and *P. aeruginosa*, which account for 20% and 24% of VAP respectively [44]. *Pseudomonas aeruginosa* can neither bind nor kill yeast-forms of *C. albicans*, however this bacterium can form a dense biofilm on *C. albicans* filaments [45]. Significantly, *P. aeruginosa* virulence factors associated with human disease (*e.g.* secreted phospholipase C) have also been linked with biofilm formation and killing of the filamentous forms of *C. albicans* which might then provide nutrients for bacterial biofilm development [45]. Colonisation of the respiratory tract with *C. albicans* is associated with an increased risk of *Pseudomonas* VAP [46], and conversely antifungal treatment of patients demonstrating airway colonisation with *Candida* spp. have less *P. aeruginosa* VAP [47]. An anti-candidal approach has also been successful in reducing the biofilm on laryngeal prosthetic devices [48]. The variation in aerobic bacterial counts between patients ranged between 0 and 2.1×10^8^ cfu/cm^2^. The reason for such variation (including the absence of bacterial growth in 5 endotracheal tubes) could in part relate to the effectiveness of the seal between endotracheal tube cuff and mucosal wall of the trachea. A totally effective seal would prevent leakage of pooled secretions from above the cuff leading to subsequent contamination of the endotracheal tube lumen and lungs. The effectiveness of the seal will vary with pressure variation between the balloon of the cuff and by the extent of ‘folding’ in the cuff material. Micro-channels generated by such folds can serve as conduits for microbial passage. Oral hygiene parameters of the patients were not assessed in this study and it may be the case that patients with poorer oral hygiene exhibited higher endotracheal tube contamination and *vice versa*. Some loss of microbial viability between the time of removal of the endotracheal tube from the patient and its subsequent processing for culture is likely, despite the time between these procedures being kept as short as possible. The fact that microbial DNA was obtained from these endotracheal tubes would suggest microbial cells (viable or non-viable) had been present and highlights the benefit of supplementing the culture analysis with molecular investigation.

The present study has shown that DGGE can be used to generate community profiles of the microflora associated with endotracheal tubes. This non-cultural approach has revealed significant microbial diversity with between 3 and 22 (mean 6) bands present per sample. These results are similar to those of other studies of microbial communities assessed by non-cultural methods. In the majority of systems studied, molecular methods detect the presence of a greater number of species than culture alone. For example, in the analysis of failed root canal lesions, between one and 26 bands (mean six) were detected by molecular methods compared to 3-4 isolates detected by culture [49].

There is no direct correlation between the number of bands detected by DGGE and the number of species present in a sample since numerous factors affect band number including sampling, PCR and differential DNA extraction biases, heteroduplex formation and divergence within multicopy number rDNA families. However, following excision and sequence analysis of individual bands present on DGGE gels, it has been reported that on average each band represents not just one, but an average of 2.7 phylotypes or bacterial species [50]. Therefore, the species richness within the endotracheal tube may be even greater than that implied in the current study.

Previous studies of complex human microbial communities using DGGE have revealed similar levels of diversity to that encountered in the present study. In a study of the microflora associated with dental abscesses from two different geographical locations a total of 99 distinct bands were detected [51]. In both this and an earlier study of root canal lesions many of the observed bands were shared between patient profiles [49]. Although a small number of shared bands were observed in our current study, the inter-patient profile diversity was generally greater when compared to previous studies. Moreover, there was significant intra-patient diversity for the three patients from whom more than one endotracheal tube community profile was obtained. The marked diversity revealed is perhaps not surprising given that the oropharyngeal microflora is considered to be the most complex in the human body with more than 700 different species present of which over 50 % have not yet been cultivated [17].

Of the numerous culture-independent approaches that are available, DGGE has proven useful in the analysis of many different microbial communities, both environmental and those associated with human infection. The principle advantage is the ability to analyse those bacteria which are either difficult to culture or which have not yet been cultured. The presence in bronchoalveolar lavages (BALs) of both novel microorganisms and those that are already ‘known’ on the basis of sequence characterisation but have not yet been cultivated, has been demonstrated [52]. This research employed a ribosomal RNA cloning and sequencing approach which provided valuable information on the microbial composition of BALs obtained from traumatic ICU patients. The approach demonstrated considerable diversity of bacterial types present in BAL samples with over 54 different bacterial types present, of which 38 had not previously been detected at this site.

Both DGGE and the cloning/sequencing approach have a role to play in the analysis of complex microflora including VAP associated bacteria. DGGE is less technically demanding, less labour intensive and more affordable. Consequently, it may be used for the simultaneous analysis of multiple samples, facilitating the direct comparison of bacterial communities from numerous sources and the effects of interventions in longitudinal studies. DGGE has been used successfully to demonstrate differences in profiles between two distinct patients groups, both in geographic and clinical contexts. The method has been used to demonstrate that both the diversity and complexity of the plaque microflora was less in children with caries compared to caries free children [50]. Differences in DGGE profiles of dental abscesses of Brazilian and USA origin have also been shown [51]. In contrast to many other profiling techniques, DGGE has the advantage that if marker signatures or bands appear to be significant, the bands can be excised and sequenced for further characterisation. In this way, a better characterisation of the microflora associated with dental caries has been demonstrated [51]. Furthermore, as in the current study, the technique can incorporate marker profiles of species of interest [53].

Care must however be taken if DGGE is used as a technique for species identification as it does have severe limitations in this regard. Since sequences from different species can co-migrate to the same position in the gel [54] identification based on excised band sequencing requires every fragment to be singularised before sequencing. This can involve either another gradient gel electrophoresis step or a time-consuming cloning process [55]. Furthermore if comparison is made with specific PCR analysis then consideration has to be made to the lower sensitivity of DGGE when compared to PCR directed against a specific microorganism [53]. It has been reported that the sensitivity of DGGE in a mixed community is about 10^3^ cells [56]. However, in terms of community profiling this may not necessarily be a disadvantage since DGGE will reflect the predominant microorganisms present and in the field of medical microbiology, numerical predominance is a fundamental principle.

In summary, a combination of PCR, molecular profiling and culture was used to ascertain the bacterial diversity and presence of specific marker microorganisms in endotracheal tube biofilms. In this context, molecular profiling using DGGE demonstrated considerable biofilm compositional complexity and inter-patient diversity and, as a method that facilitates the study of shifts in the ecological balance of biofilms, will prove invaluable in future longitudinal or interventional studies. We hypothesised that oral microorganisms form part of the endotracheal tube biofilm and, indeed, demonstrated that these bacteria were present. This observation offers the possibility of alternative therapeutic strategies, not necessarily based on anti-infective agents, for preventing VAP which may include mechanically reducing oral plaque, the targeting of quorum sensing signal molecules [57], [58] or modulation of the oral microflora [59].
